# [Corrigendum] Proliferation, migration and invasion of triple negative breast cancer cells are suppressed by berbamine via the PI3K/Akt/MDM2/p53 and PI3K/Akt/mTOR signaling pathways

**DOI:** 10.3892/ol.2026.15639

**Published:** 2026-05-06

**Authors:** Lili Liu, Jiadong Yan, Ying Cao, Yan Yan, Xiang Shen, Binbin Yu, Li Tao, Shusheng Wang

Oncol Lett 21: 70, 2021; DOI: 10.3892/ol.2020.12331

Following the publication of the above article, an interested reader drew to the authors’ attention that the ‘BBM 10 μM/Transwell’ and ‘Ctrl/Matrigel transwell’ data panels in Fig. 4C on p. 6, showing the results of Transwell migration and invasion assays, were apparently identical. Furthermore, upon performing an independent analysis of the data in this paper in the Editorial Office, it came to light that the ‘BBM 20 μM/0 h’ and ‘BBM 40 μM/0 h’ data panels for the MCF-7 cell line in Fig. 4B also contained an overlapping section, such that data which were intended to show the results of differently performed scratch-wound assay experiments had apparently been derived from the same original source.

After having re-examined their original data, the authors have realized that these figure parts contained data which were inadvertently selected incorrectly for Fig. 4. The revised version of Fig. 4, now showing the correct data for the ‘BBM 40 μM/0 h’ data panel for the MCF-7 cell line in Fig. 4B and the ‘Ctrl/Matrigel transwell’ data panel in Fig. 4C, is shown on the next page. Note that the errors made during the assembly of this figure did not affect the overall conclusions reported in the paper. All the authors agree with the publication of this corrigendum. The authors are grateful to the Editor of *Oncology Letters* for allowing them the opportunity to publish this, and also apologize to the readership for any inconvenience caused.

## Figures and Tables

**Figure 4.**
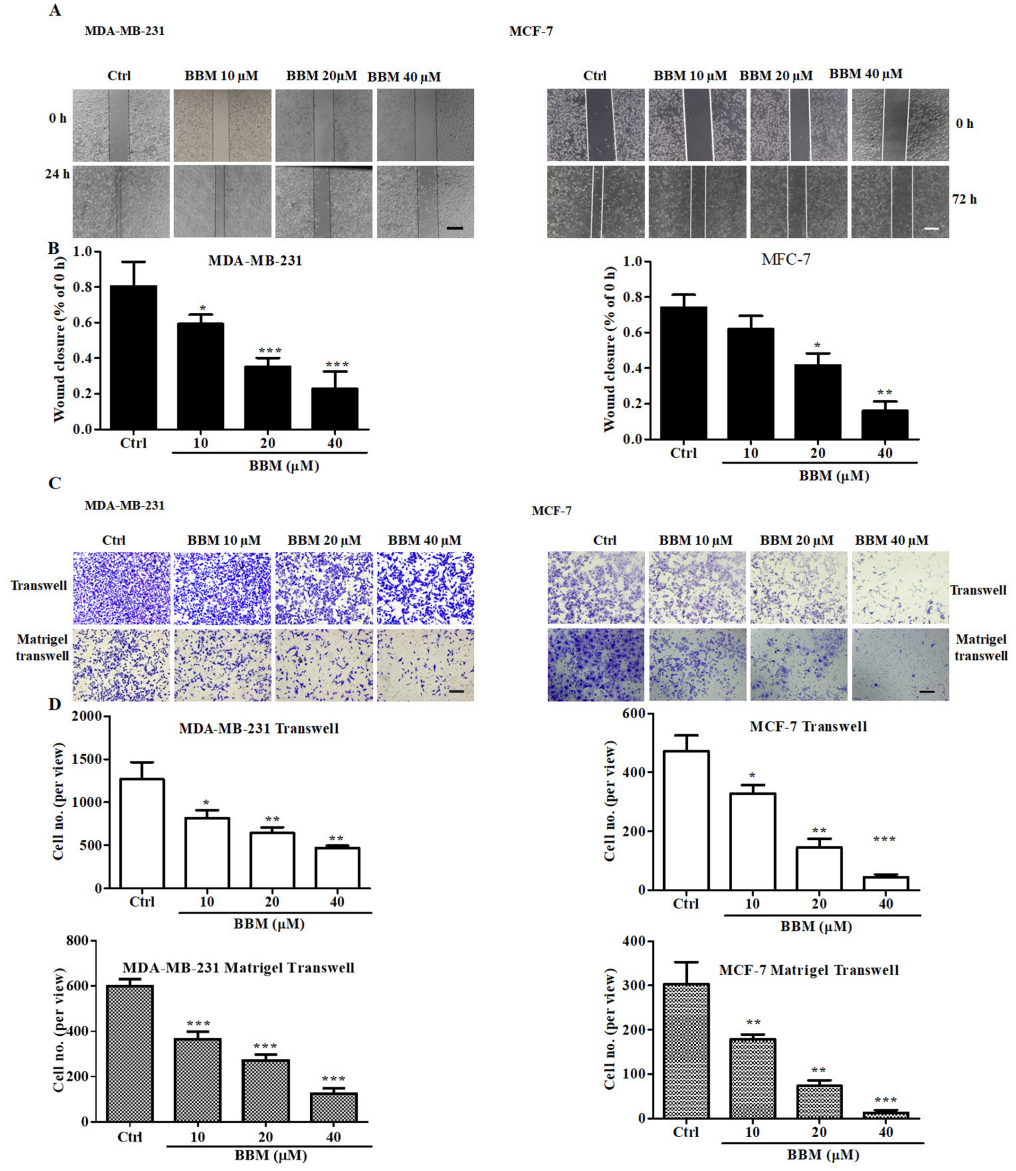
BBM inhibits the migration and invasion of triple negative breast cancer cells. Cells were treated with BBM (10, 20 and 40 µM), migration and invasion were tested using (A) wound closure assays (scale bar, 100 µm) and (B) quantified. (C) Transwell and Matrigel transwell assay cell images (scale bar, 100 µm) and (D) quantification of the results. The Ctrl group was untreated cells. Data are presented as the mean ± standard deviation (n=3). *P<0.05, **P<0.01, ***P<0.001 vs. Ctrl group. BBM, Berbamine; Ctrl, control..

